# Anesthetic Propofol-Induced Gene Expression Changes in Patients Undergoing Coronary Artery Bypass Graft Surgery Based on Dynamical Differential Coexpression Network Analysis

**DOI:** 10.1155/2016/7097612

**Published:** 2016-06-29

**Authors:** Da Yu, Li-Jun Huang, Na-Mi Chen

**Affiliations:** Department of Anesthesiology, Yinzhou Hospital Affiliated to Medical School of Ningbo University, Ningbo, Zhejiang 315000, China

## Abstract

We aimed to determine the influence of anesthetic propofol on gene expression in patients treated by coronary artery bypass graft (CABG) surgery based on differential coexpression network (DCN) and to further reveal the novel mechanisms of the cardioprotective effects of propofol. Firstly, we constructed the DCN for disease condition based on Pearson correlation coefficient (PCC) and weight value. Secondly, the inference of modules was applied to search modules from DCN with same members but varied connectivity. Furthermore, we measured the statistical significance of the modules for selecting differential modules (DMs). Finally, attract method was used for DMs analysis to select key modules. Based on the *δ* value, 11928 edges and 2956 nodes were chosen to construct DCNs. A total of 29 seed genes were selected. Moreover, by quantifying connectivity changes in shared gene modules across different conditions, 8 DMs with higher connectivity dynamics were identified. Then, we extracted key modules using attract method, there were 8 key modules, and the top 3 modules were module 1, 2, and 3. Furthermore, GCG, PPY, and PON1 were initial seed genes of these 3 key modules, respectively. Accordingly, GCG and PON1 might exert important roles in the cardioprotective effects of propofol during CABG.

## 1. Introduction

Heart disease is a common, costly, and potentially fatal condition. In developed countries, around 2% of adults have heart disease and in those over the age of 65, this increases to 6–10% [1]. At present, with increasing numbers of patients, coronary artery bypass grafting (CABG) has become the most common operation in the world [2]. In order to provide organ protection, the doctors will perform the preoperative anesthesia. Propofol (2,6-diisopropylphenol) is an intravenous sedative agent widely used for anesthesia and sedation [3]. Propofol has antioxidant and antiapoptotic effects [4–6]. In critically ill patients, propofol has been found to be superior to some medicine in both effectiveness and overall cost [7]. At present, it has been verified that propofol can promote the recovery after surgery [8]. Moreover, it has been suggested that propofol attenuates myocardial lipid peroxidation during CABG surgery [9]. Growing evidences have indicated that propofol has effective cardioprotective effects, yet little is known about how propofol is to protect the heart cells from molecular biology aspect during the CABG.

During the past decades, rapid advances in high-throughput technologies have brought unprecedented opportunities for the large-scale analysis of the disease molecular mechanisms. Moreover, network biology has demonstrated to be a powerful tool for analyzing complex molecular networks to identify informative genes which exerts important functions in the development and progression of disease [10, 11]; for example, coexpression networks are employed in most researches. However, the drawback of coexpression networks may reduce the statistical power to screen pathways which are abnormal in disease conditions. Furthermore, too big network possibly neglects a certain number of important genes and interactions [12], and assessing modules or subnetworks of the intricate network can avoid this kind of difficulty [11, 13]. In small module or subnetwork, functions of individual genes and gene-gene interactions may be studied in more detail and precisely [14]. Traditionally, if one gene-gene interaction between a gene pair exhibits highly correlated strength in one state, this interaction will be chosen as an edge of network [15]. Nevertheless, if one gene in the interaction is differently expressed but the other one is not, it may not be regarded as significant interaction for the whole dataset. To a great degree, the difficulty can be solved via constructing differential coexpression network (DCN). Fortunately, Ma et al. [16] offered the module-search method to screen coherently differentially expressed gene modules with common members yet varied connectivity in DCN. Moreover, differential expression network has been created to identify groups of genes that exhibit coherent differential activities between healthy and diseased conditions [17]. In addition, Li and colleagues used multiple differential expression networks to extract significant genes in rectal cancer [18]. Zhai et al. also used DCN to identify the biomarkers and pathway-related modules in ovarian cancer based on topological centralities [19].

In accordance with the reports in cardioprotection during CABG surgery after propofol treatment, we hypothesized that propofol would elicit genomic responses to cardiac surgery in human hearts. Significantly, gene expression changes were related with clinically crucial cardiovascular biosignatures and to physiologic parameters of cardiac function. The key innovation of this novel method can extract unique and shared modules from differential expression networks. By definition, sets of genes that are differentially expressed in diseased condition but do not show correlated expression pattern will not be identified as a differentially coexpressed gene module [20]. Thus, the dysregulation of differential module (DM) gives more proof to the molecular mechanism of cardioprotective effects of propofol during CABG.

In this paper, in order to explore the dynamics changes in atrial gene expression induced by propofol, we utilized the modules-search algorithm to explore the important modules based on the DCN. Firstly, a dataset from the European Bioinformatics Institute (EMBL-EBI) database (E-GEOD-4386) was downloaded. DCN construction was implemented, followed by the identification of modules via three major steps of seed genes selection, module search by seed expansion and entropy minimization, and refinement of modules. Then, statistical significance of modules was computed to select the differential modules (DMs). Such modules might be helpful to reveal the molecular mechanisms of the cardioprotective effects of propofol during CABG.

## 2. Materials and Methods

### 2.1. Expression Profile Data Collection

The gene expression profile of E-GEOD-4386 [21] was recruited from the platform of A-AFFY-44-Affymetrix GeneChip Human Genome U133 Plus 2.0 [HG-U133_Plus_2] of the EMBL-EBI database which was recommended by most journals as a repository for data supporting peer-reviewed publications [22]. The 10 atrial samples were collected from patients undergoing CABG surgery with anesthetic propofol treatment, named as propofol group. Moreover, 10 control samples comprised the same patients prior to CABG surgery, determined as baseline group. Through the mapping between each probe and the corresponding official symbol by getSYMBOL, we got one expression profile data which contained 20389 genes.

We downloaded the human protein-protein interaction networks (PPIN) comprising 787896 interactions from the database Search Tool for the Retrieval of Interacting Genes/Proteins (STRING, http://string-db.org/) which provided a comprehensive, yet quality-controlled collection of protein-protein associations for a large number of organisms [23]. Meanwhile, the expression profile data was mapped to the PPIN and a new PPIN was obtained.

### 2.2. Construction of DCN

For this disease, the DCN construction was made up of two steps. Firstly, a binary coexpression network was constructed. Then, edge weight was assigned on the basis of differential gene expression between baseline and propofol group.

In this research, we firstly calculated the absolute value of Pearson correlation coefficient (PCC) for these interactions in propofol group. The interactions whose correlations were greater than the predefined threshold *δ* (*δ* = 0.9) were selected to construct the coexpression network.

In addition, edge weights were assigned in the binary coexpression network based on the *P* value of differential gene expression between two conditions. In this paper, we applied EdgeR [24] to calculate the weight value. The formula was as follows:

(1) $$\begin{array}{c} {\text{w}}_{i,j}=\left\{\begin{array}{cc} \frac{{\left(\log P_{i}+\log{\text{P}}_{\text{j}}\right)}^{\text{1}/\text{2}}}{{\left(\text{2}*\max_{l\in V}\left|\log{\text{P}}_{\text{l}}\right|\right)}^{\text{1}/\text{2}}}, & \text{if }\text{cor}\;\left(i,j\right)⩾\delta ,\\ 0, & \text{if }\text{cor}\;\left(i,j\right)<\delta , \end{array}\right. \end{array}$$

where *P*_*i*_ and *P*_*j*_ were *P* values of differential expression genes *i* and *j*, respectively. *V* was the gene set of the coexpression network, and cor⁡ (*i*, *j*) stood for the absolute value of PCC between genes *i* and *j*. The genes with higher weights likely participated in a pathway that exhibited differential activities between the two conditions being compared.

### 2.3. Identification of Modules in DCN

Module is designed for identifying gene modules with common members but varied connectivity across multiple molecular interaction networks [25]. Here we applied the modules algorithm to identify modules. Specific steps were as follows.

Firstly, we performed the seed prioritization for finding the seed genes. Here we ranked the genes in DCN by the topological feature of the gene (degree) and calculated the importance of each gene* i*. The algorithm was defined as

(2) $$\begin{array}{c} g\left(\;i\;\right)=\underset{{y\in N}_{k}\left(i\right)}{\sum }A_{ijk}^{\prime }g\left(\;j\;\right), \end{array}$$

where *N*_*k*_(*i*) was the set of neighbors in each network *G*_*k*_; *A*_*k*_′ represented the degree normalized weighted adjacency matrix which was calculated as *A*_*k*_′ = *D* ^−1/2^ *A*_*k*_ *D* ^1/2^, where *D* was diagonal matrix with *A*_*k*_. We calculated a* z*-score for each *g*(*i*) and then ranked the genes by averaging the *z*-scores across all networks. Ultimately, the top 1% genes in the network were selected as the seed genes.

Secondly, module search was implemented according to the seed genes expansion and entropy minimization. For the defined seed gene *v* ∈ *V*, we treated it as a module *C* in the network. After that, we added the gene *u* which was the neighbor of gene *v* into the module *C* and obtained the new module *C*′. We defined the entropy change Δ*H* as the connectivity of the two modules. The function was defined: Δ*H*(*C*′, *C*) = *H*(*C*) − *H*(*C*′).

When Δ*H*(*C*′, *C*) > 0, it represented that the addition of gene *u* improved the connectivity of the module *C*. Similar to the above, we joined all the neighboring gene *u* with Δ*H*(*C*′, *C*) > 0 into the module *C* till Δ*H* was not increased.

Thirdly, the modules were refined. In this step, we removed the modules with the number of nodes < 5. In addition, to merge overlapping candidate modules, we employed Jaccard index [26] which is the ratio of intersection over union for two sets. In our study, two candidate modules were merged when the Jaccard index was 0.5.

### 2.4. Analysis of Statistical Significance of Modules

In the current study, we calculated the statistical significance of modules according to the null score distribution of modules generated by random networks. Specific steps were as follows: each randomized network which was made up of edges captured from interactions in DCN was identified, and the number of edges in randomized network was the same as that in DCN. After constructing 100 times randomly for each network, we implemented modules search on the randomized networks to obtain the null distribution of module scores. Next, on the basis of the null distribution, we evaluated the *P* value of each module as the probability of the module having smaller score by chance. Moreover, we used the Benjamini-Hochberg method to correct the *P* values [27]. Finally, we defined the modules with *P* value ≤ 0.05 as the DMs.

### 2.5. Detection of the Key Modules by Attract Method

Attract is essentially the inverse of more traditional gene expression analysis approaches. In this paper, we used attract method [28] to identify the key modules of propofol on myocardial cell based on the above DMs. On the basis of GSEA-ANOVA, an ANOVA model [28] was fitted to each gene and the expression of one gene was regulated by a single factor standing for the groups as different levels of this class. According to the ANOVA model, the *F*-statistic value for gene *m* is counted:

(3) $$\begin{array}{c} F^{\left(m\right)}=\frac{{MSS}_{m}}{{RSS}_{m}}, \end{array}$$

where MSS_*m*_ is the mean treatment sum of squares and captures the amount of variation because of the group-specific effects:

(4) $$\begin{array}{c} {MSS}_{m}=\frac{1}{K-1}\overset{K}{\underset{k=1}{\sum }}r_{k}{\left[\;y_{\cdot k}^{\left(m\right)}-y_{\cdot \cdot }^{\left(m\right)}\;\right]}^{2}. \end{array}$$

RSS_*m*_ represents the residual sum of squares, and it is calculated using the following formula:

(5) $$\begin{array}{c} {RSS}_{m}=\frac{1}{N-K}\overset{K}{\underset{k=1}{\sum }}\overset{r_{n}}{\underset{\;n=1}{\sum }}{\left[\;y_{nk}^{\left(m\right)}-y_{\cdot \cdot }^{\left(m\right)}\;\right]}^{2}, \end{array}$$

where *N* means the total number of samples, and the overall mean is counted:

(6) $$\begin{array}{c} y_{\cdot \cdot }^{\left(m\right)}=\frac{1}{K}\overset{K}{\underset{k=1}{\sum }}\left(\;\frac{1}{r_{k}}\overset{r_{k}}{\underset{n=1}{\sum }}y_{nk}^{\left(m\right)}\;\right). \end{array}$$

Here, large value of the* F*-statistic suggested a strong group-specific expression change. A small* F*-statistic was in a similar way. Next,* t*-test was performed for the log_2_-transformed* F*-statistics values from above the modules. Moreover, Benjamini-Hochberg-based method was utilized to adjust the *P* values [27]. In our analysis, the modules with adjusted *P* values ≤ 0.05 were considered as key modules.

### 2.6. Pathway Enrichment Analysis

The occurrence of diseases is often induced by the dysregulation of pathways involved in the biological process. Because of this, we focused on the pathways enrichment analysis for key module genes to narrow down our analysis. Kyoto Encyclopedia of Genes and Genomes (KEGG) database provides a reference knowledge for understanding biological processes via pathway aligning, which is to align genes to reference pathways to deduce the behaviors of cell [29]. In our study, all reference pathways were downloaded from KEGG database. Then, genes in key modules were mapped to the reference pathways to identify the abnormal pathways. Significant pathways of the genes in key modules between two groups were identified when the FDR was set as 0.01 and the number of genes in pathways was not less than 10.

## 3. Results

### 3.1. Construction of DCN

In this paper, a total of 787896 humans PPIs (16730 genes) were downloaded from STRING database. After the genes in microarray data were aligned to the ensemble PPIN, a new PPIN including 15171 genes and 728200 interactions was obtained. In order to construct coexpression network, edges were extracted according to the absolute value of the PCC of the expression profiles of two genes. Based on the absolute value > 0.9, we selected 11928 interactions and 2956 genes to construct the DCN.

### 3.2. Identification of Modules in DCN

Based on* z*-score of each gene, we selected a total of 29 seed genes in DCN. The result was shown in Table 1. Among these 29 seed genes, there were 4 genes with* z*-score value > 110, for example, CSN1S1 (*z*-score = 139.75), ALB (*z*-score = 130.93), ALPP (*z*-score = 120.37), and CDH1 (*z*-score = 113.58). Subsequently, taking these 29 seed genes as start, we implemented module identification based on the entropy decrease Δ*H*(*C*′, *C*). Moreover, according to the entropy change, we showed that gene modules with higher connectivity dynamics were under Δ*H*(*C*′, *C*) > 0. In this research, we obtained 8 modules after removing the modules with the number of nodes < 5 as well as merging into one module if the Jaccard index ≥ 0.5 between the two modules.

### 3.3. Analysis of Statistical Significance of Modules

After constructing 100 times randomly for each network, we searched 5845 modules in all. Through the calculation and correction of the *P* value of all modules, at a *P* value threshold of 0.05, we screened several modules as DMs. What was more, we noticed that all the above 8 modules were DMs owing to their *P* value < 0.05 after the statistical analysis. The result was shown in Table 2.

### 3.4. Detection of the Key Modules by Attract Method

**Figure 1:**
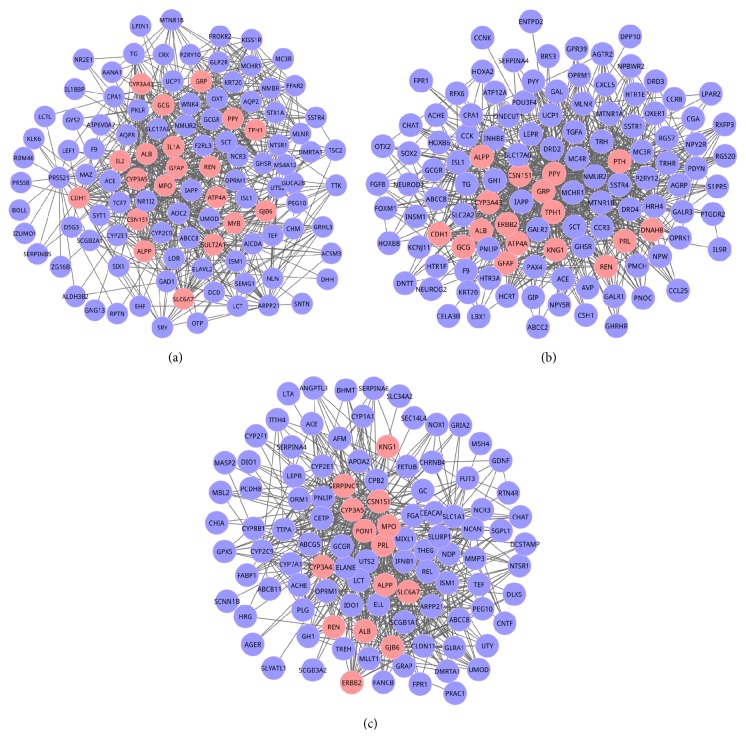
The 3 key modules identified in the differential coexpression network (DCN) of baseline and propofol group based on the adjusted *P* values by attract method. (a), (b), and (c) represented module 1, module 2, and module 3, respectively. Nodes were genes, and edges were the interactions among two genes. There were 119 nodes and 866 edges in module 1; 112 nodes and 766 edges were contained in module 2; module 3 consisted of 105 nodes and 649 edges. The pink nodes were seed genes.

In this part, we evaluated the adjusted *P* values for these 8 DMs utilizing the attract way. We found that these 8 DMs were key modules. In order to further verify the connectivity dynamics of two genes in key modules, we focused on the weight distribution of edges (Table 3). Significantly, we found that a majority of interactions distributed in the range of 0.1–0.2, which indicated that genes were connected with each other closely, and these key modules had good connectivity properties in propofol group. Here, the top 3 modules were module 1 (adjusted *P* value = 1.12*E* − 11), module 2 (adjusted *P* value = 3.43*E* − 09), and module 3 (adjusted *P* value = 6.21*E* − 09). Meanwhile, among the 3 key modules, GCG, PPY, and PON1 were initial seed genes, respectively. Here we showed the 3 key modules in Figure 1.

### 3.5. Pathway Enrichment Analysis

To further research the dysregulated biological functions in disease condition, pathway analysis was conducted based on the genes in key modules. Based on FDR < 0.01 and the number of genes ≥ 10, genes in key modules 1, 2, 3, 5, 6, and 7 were simultaneously enriched in the pathway of neuroactive ligand-receptor interaction. The gene count and the FDR were shown in Table 4.

## 4. Discussion

It has been reported that anesthetic modulates gene expression [30] and provided organ protection [31]. Propofol is a general anesthetic widely used for the induction and maintenance of anesthesia during cardiac surgery and for postoperative sedation. Furthermore, several researches have previously published that propofol conferred protection against damage to the myocardium for patients undergoing CABG [32–34]. However, the related protection mechanisms from the molecular dynamics level were rarely involved. This study aimed at identifying myocardial transcriptional phenotypes to predict dynamics variation of the biomarkers and function in myocardial cell taking propofol anesthesia undergoing CABG surgery. In our research, the key innovation is the ability to identify several important modules from DCN, each of which representing a different perturbation condition. Here we noticed that genes in 8 DMs had more connections and located in more central positions in the network. Particularly, the 3 key modules had higher correlation with the disease phenotype. What is more, among the 3 modules, GCG, PPY, and PON1 were initial seed genes, respectively. These data underlined the importance of these genes and their corresponding modules for the research of dynamic variation in myocardial cell induced by propofol.

GCG (glucagon) is a protein coding gene, which plays a key role in glucose metabolism and homeostasis. It has been reported that the secretion of glucagon-like peptide-1 and glucose-dependent insulinotropic polypeptide is under influence of the digestion and absorption of nutrients in the small intestine and that pancreatic enzyme substitution increased insulin secretion [35]. Concurrently, Jeppesen et al. [36] have demonstrated that glucagon-like peptide-2 treatment reduces fecal weight and enabled short bowel syndrome patients to maintain their intestinal fluid. Subsequent researches have shown that glucagon-like peptide-1 infusion improved regional and global left ventricular function in patients with acute myocardial infarction and severe systolic dysfunction after successful primary angioplasty [37]. Significantly, Kitamura et al. [38] have indicated that propofol contributes to the stable glucose metabolism during surgery. In light of these, we infer that propofol might play important roles in cardioprotective effects, at least in part, through regulating GCG expression.

Serum paraoxonase 1 (PON1) also known as A esterase, homocysteine thiolactonase, or serum aryldialkylphosphatase 1 is an enzyme that in humans is encoded by the PON1 gene [39]. Decades of research have indicated that PON1 protected humans from the acute and chronic harmful effects of a variety of disease. For example, it has been verified that PON1 is also a major antiatherosclerotic component of high-density lipoprotein [40, 41]. Li et al. [42] suggested that subjects of low PON1 activity may be more susceptible to arsenic-related cardiovascular disease. Moreover, significant decrease of PON1 activity confirmed the high risk of cardiovascular diseases in smokers [43]. What is more, the evaluation of PON1 activity demonstrated its decrease was a risk factor associated with increased coronary heart disease susceptibility [44, 45]. In this research, compared with the control cases without propofol after CABG surgery, we investigated the disease group received the intravenous anesthetic propofol after undergoing CABG surgery and showed the crucial gene PON1 in 3-DM. Hence, it provided conclusive evidence on the effects of propofol on heart disease by altering the activity of PON1.

Significantly, in the current study, pathway enrichment results indicated that genes in key modules 1, 2, 3, 5, 6, and 7 were simultaneously enriched in the pathway of neuroactive ligand-receptor interaction. As documented, the pathway of neuroactive ligand-receptor interaction has been demonstrated to be associated with the development and progress of cardiovascular diseases [46]. The key proteins in this pathway, such as angiotensin, adrenergic, and calcitonin receptor-like neurotensin receptors, have also been indicated to be closely related to cardiac function [47, 48]. Recently, growing evidence has suggested that propofol exerts direct inhibitory effects on adrenergic receptor signal transduction in cardiomyocytes [49]. Accordingly, we infer that these key modules might be beneficial to reveal the cardioprotective effects of propofol during CABG.

## 5. Conclusion 

Taken together, these key modules and their seed genes might exert important roles in the cardioprotective effects of propofol. However, the identified modules and their initial seed genes in this study were not confirmed in animal models. Further study in animal models may be required in the later work.

## Figures and Tables

**Table 1:** The 29 seed genes identified in the differential coexpression network (DCN) and the distribution of their *z*-scores.

Seed genes	*z*-score
CSN1S1	139.75
ALB	130.93
ALPP	120.37
CDH1	113.58
IL2	107.69
ERBB2	99.76
IL4	94.42
CYP3A43	94.21
MPO	90.49
ATP4A	86.75
PPY	82.40
GFAP	77.59
SLC6A7	76.27
CYP3A5	75.42
GRP	75.17
GCG	73.66
IL1A	72.32
DNAH8	71.73
PON1	71.38
NANOG	70.21
PTH	67.75
KNG1	66.82
SERPINC1	66.70
PRL	66.12
REN	66.08
TPH1	63.74
GJB6	62.07
MYB	61.89
SULT2A1	61.44

**Table 2:** The 8 differential modules (DMs) with adjusted *P* values < 0.5 and the initial seed genes in the DMs.

Modules	Adjusted *P* values	Initial seed gene
Module 1	1.12*E* − 11	GCG
Module 2	3.43*E* − 09	PPY
Module 3	6.21*E* − 09	PON1
Module 4	1.32*E* − 07	IL1A
Module 5	2.59*E* − 07	GJB6
Module 6	7.97*E* − 06	GRP
Module 7	7.97*E* − 06	TPH1
Module 8	2.23*E* − 05	SULT2A1

**Table 3:** The distribution of weight values of 8 key modules.

Modules	Number of interactions	Number of interactions	Number of interactions
	0–0.1	0.1–0.2	0.2–0.3
Module 1	0	865	1
Module 2	1	764	1
Module 3	1	645	3
Module 4	1	742	0
Module 5	1	531	3
Module 6	0	731	1
Module 7	4	441	1
Module 8	3	693	1

**Table 4:** Kyoto Encyclopedia of Genes and Genomes (KEGG) pathway enrichment analysis of genes in key modules based on false discovery rate (FDR) < 0.01 and gene count ≥ 10.

Modules	KEGG pathways	Gene count	FDR
Module 1	Neuroactive ligand-receptor interaction	15	8.32*E* − 06
Module 2	Neuroactive ligand-receptor interaction	37	7.10*E* − 26
Module 3	Neuroactive ligand-receptor interaction	10	1.63*E* − 03
Module 4	Cytokine-cytokine receptor interaction	29	6.91*E* − 16
	JAK-STAT signaling pathway	20	1.73*E* − 11
	Toll-like receptor signaling pathway	11	4.32*E* − 05
	Natural killer cell mediated cytotoxicity	12	6.04*E* − 05
Module 5	Neuroactive ligand-receptor interaction	10	3.76*E* − 03
Module 6	Neuroactive ligand-receptor interaction	33	4.62*E* − 25
Module 7	Neuroactive ligand-receptor interaction	16	2.11*E* − 07
Module 8	Steroid hormone biosynthesis	11	4.36*E* − 09
